# Exogenous Glutathione Enhances Salt Tolerance in Patchouli by Promoting the Antioxidant Capacity and Activating the Flavonoid Biosynthesis Pathway

**DOI:** 10.3390/plants15030457

**Published:** 2026-02-02

**Authors:** Heqin Yan, Yating Su, Jieyun Fang, Muhammad Zeeshan Ul Haq, Weizhe Su, Yougen Wu, Jiangtao Hu, Ya Liu

**Affiliations:** 1 School of Breeding and Multiplication (Sanya Institute of Breeding and Multiplication), School of Tropical Agriculture and Forestry, Hainan University, Sanya 572025, Chinadrzeeshanulhaq@gmail.com (M.Z.U.H.);; 2 Institute of Urban Agriculture, Chinese Academy of Agricultural Sciences, Chengdu National Agricultural Science and Technology Center, Chengdu 610213, China; hujiangtao@caas.cn; 3 Key Laboratory for Quality Regulation of Tropical Horticultural Crops of Hainan Province, School of Tropical Agriculture and Forestry, Hainan University, Haikou 570228, China

**Keywords:** *Pogostemon cablin*, glutathione, antioxidation, salt stress, flavonoid

## Abstract

Salt stress is a severe threat to medicinal plants, adversely affecting their growth, yield, and quality. As a key antioxidant tripeptide, glutathione (GSH) confers salinity stress resilience in plants. However, how GSH shapes the plant tolerance to salt stress remains a mystery, especially in medicinal plants, including *Pogostemon cablin*. In this study, we investigated the regulatory effects of exogenous GSH on *P. cablin* seedlings under salt conditions. The results showed that GSH significantly improved seedling quality under both normal and salt conditions, evidenced by the increased shoot and root dry weight, plant height, and root length. Moreover, GSH effectively protected the photosynthetic system against salt-mediated damage via raised chlorophyll a, chlorophyll b, carotenoids, quantum yield of photosystem II [Y (II)], and PSII maximum efficiency (Fv/Fm). Furthermore, GSH stimulated the antioxidant defense system, including GSH, AsA, SOD, CAT, APX, POD, and GR, to restrain salt-induced malondialdehyde production and ROS burst. In addition, GSH treatment promoted the biosynthesis of secondary metabolites, including total polyphenol and flavonoid. RNA-seq analysis revealed that the activation of the flavonoid biosynthetic pathway significantly enhanced salt tolerance in *P. cablin*. Notably, several key regulatory genes within this pathway, including *PAL*, *4CL*, *C4H*, *CHI*, *ANS*, *F3′H*, and *CYP93*, were significantly upregulated 24 h following GSH application under salt conditions. Therefore, exogenous GSH alleviates salt-induced oxidative stress in *P. cablin* via enhancing the antioxidant defense system and flavonoid biosynthetic activation. These findings provide valuable insights into the dual defense strategies of GSH for conferring salt resistance in plants.

## 1. Introduction

*Pogostemon cablin* (Blanco) Benth., commonly known as patchouli, is a distinguished member of the Lamiaceae family with considerable significance in traditional Chinese medicine (TCM), as well as applications as a spice and a homologous food-medicine plant in China [1]. Predominantly cultivated in the regions of Guangzhou and Hainan provinces, China, patchouli is esteemed for its therapeutic attributes, including its aromatic, damp-resolving, appetite-enhancing, antiemetic, and heat-clearing properties [1,2]. Beyond its integration into over 30 TCM formulations, patchouli is a fixative and blending agent in premium perfumes and fragrances and a food additive, underscoring its multifaceted utility [2,3]. Despite its pharmaceutical prominence and commercial value, patchouli cultivation encounters significant challenges due to rising soil salinity [4,5], a constraint particularly pronounced in Hainan Province, China. Long-term adjacent to the sea, Hainan suffers from incomplete freshwater leaching of soil salts due to the short history of reclamation. According to reports, the soil salinity ranges from 3.9‰ to 13.10‰ in Haikou, Hainan Province [6]. Coupled with intense evaporation, salts accumulate on the soil surface, which severely impairs crop growth. In recent years, due to reasons such as accelerated urbanization in coastal areas, widespread use of chemical fertilizers and pesticides, and unreasonable irrigation methods, along with global climate warming, the area of salt-affected soil has been expanding continuously [7,8]. Salt stress markedly impairs patchouli’s morphological development and growth, substantially reducing yield and quality [4,5]. Consequently, developing and cultivating salt-tolerant varieties is imperative to ensure sustainable, high-quality advancements in the patchouli industry.

Salt stress is a pivotal factor affecting crop quality and productivity [9]. Excessive sodium ion (Na^+^) uptake by plant roots from soil substrates significantly disrupts developmental and metabolic processes [10]. This stressor induces osmotic stress, ionic toxicity, and deficiencies in essential mineral nutrients while also perturbing the Na^+^:K^+^ equilibrium [10,11]. The resultant osmotic and ionic imbalances precipitate secondary stress responses within plant systems, including accumulating toxic metabolites and disrupting nutrient homeostasis. Under salt stress, plant cellular environments exhibit marked increases in reactive oxygen species (ROS) levels, such as hydrogen peroxide (H_2_O_2_), superoxide anions (O_2_^−^), and hydroxyl radicals (OH·) [10,12]. Salt stress had inhibitory effects on the growth of *P. cablin* seedlings. The higher the salt concentration was, the more obvious the inhibition effect [4,5]. Contemporary research on salt stress responses has primarily focused on model species such as *Arabidopsis thaliana* [10], *Oryza sativa* [13], and *Glycine max* [11]. In contrast, investigations into salt tolerance mechanisms in *P. cablin* remain notably scarce.

The exogenous application of phytohormones, synthetic growth regulators, or targeted chemical compounds has shown considerable promise in enhancing plant stress adaptation mechanisms [14]. In plants, glutathione exists in two principal forms: reduced (GSH) and oxidized (GSSG). The GSH is a critical intracellular antioxidant and regulatory molecule, significantly influencing various physiological processes [15]. Extensive research has demonstrated that GSH is essential in mitigating plant damage caused by diverse abiotic stresses, including salinity and heavy metal toxicity [16,17]. Exogenous GSH supplementation has upregulated key enzymes within the phenylpropanoid-flavonoid biosynthetic pathway in *C. arietinum*, enhancing isoflavonoid production [18]. Within plant systems, GSH-mediated nuclear redox regulation, through interactions with ROS, is instrumental in governing cell proliferation, developmental processes, and adaptive stress responses [19]. Multiple studies have established that GSH-mediated modulation of antioxidative systems and ionic homeostasis significantly bolsters salinity tolerance in numerous plant species [20,21]. Evidence further suggests that the exogenous application of GSH constitutes a practical approach to simultaneously enhance salt tolerance and improve productivity metrics in major crops, including *O. sativa*, *Zea mays*, and *Triticum aestivum* [21,22,23]. Under salt stress, GSH promotes growth through multifaceted protective mechanisms, such as reinforcing antioxidant defenses, scavenging ROS, preserving membrane integrity, and safeguarding photosystem II (PSII) from oxidative damage [19,24]. Despite these advances, research exploring the application of GSH in *P. cablin* remains limited, warranting further investigation to elucidate its potential to enhance salt tolerance in this species.

Flavonoids, a class of natural polyphenolic compounds, are widely distributed across various plant-derived sources, including fruits, vegetables, teas, and medicinal plants [25]. Ubiquitous in the plant kingdom, flavonoids encompass diverse subclasses such as flavones, flavanones, isoflavones, and anthocyanins [26]. Research has elucidated their multifaceted roles, including conferring resistance to abiotic stresses (cold, drought, and salinity), as well as exhibiting antioxidant, anticancer, and antitumor properties [25,27]. Contemporary studies have identified and characterized key enzymatic components of the flavonoid biosynthetic pathway across multiple plant species [13,28]. This pathway commences with chalcone synthase (CHS), which catalyzes the initial step of the metabolic cascade [26]. Subsequently, chalcone isomerase (CHI) facilitates the isomerization of naringenin chalcone into the flavanone naringenin, a critical intermediate [29]. Further diversification into compounds such as dihydroflavonols and anthocyanins occurs through the action of additional enzymes, utilizing naringenin as a substrate [29]. GSH regulates the expression of flavonoid biosynthesis-related genes through modulating the MAPK and Nrf2-ARE signaling pathways [30]. The research found that anthocyanin-related glutathione S-transferases (arGSTs) specifically catalyze the conversion of flavan-3,3,4-triol, the product of leucoanthocyanidin dioxygenase (LDOX), to anthocyanins [31]. In *P. cablin*, GSH protects the activity of flavonoid synthases by scavenging ROS, promoting the expression of key genes including CCoAOMT1-4 and DGKA1/2, and enhancing the biosynthesis capacity of flavonoids [32]. Although the biosynthetic mechanisms of flavonoids are well-documented in model plants, the regulatory influence of GSH on the gene expression and functional activity of core enzymes within this pathway remains inadequately explored, particularly in *P. cablin*. This knowledge gap underscores the need for comprehensive investigations into the physiological roles and regulatory dynamics of flavonoid compounds in patchouli plants.

While previous studies have demonstrated that exogenous GSH enhances salt tolerance in model plants and major crops (such as tomato, wheat and rice) by reinforcing antioxidant defense systems [17,21,23], critical knowledge gaps remain in medicinal plants—especially in *P. cablin*. The prior research on patchouli has focused solely on physiological responses to salt stress (such as changes in leaf ultrastructure and medicinal components [4,5]) but has not elucidated the molecular mechanisms underlying salt tolerance. To elucidate the regulatory mechanisms of GSH in mitigating salt stress, this study involved the exogenous application of GSH to *P. cablin* seedlings under both salt-stressed and control conditions. Key parameters were quantified, including growth metrics, photosynthetic pigments, and chlorophyll fluorescence. Moreover, a range of metabolites was analyzed, such as soluble protein, malondialdehyde (MDA), GSH, ascorbic acid (AsA), polyphenols, and flavonoids. The activities of antioxidant enzymes were also assessed, including SOD, CAT, APX, POD, and GR. To further explore molecular responses, comprehensive transcriptomic profiling, combined with weighted gene co-expression network analysis (WGCNA), facilitated the identification of differentially expressed genes (DEGs) critical to the stress response. Quantitative reverse transcription PCR (qRT-PCR) was utilized to evaluate the expression levels of key structural genes within the flavonoid biosynthesis pathway, including *PAL*, *4CL*, *C4H*, *CHS*, *CHI*, *F3H*, *ANS*, *F3′H*, *CYP93*, and *FLS*. These results provide foundational insights into the potential of GSH as a viable strategy for enhancing salt tolerance in patchouli.

## 2. Materials and Methods

### 2.1. Plant Materials, Experimental Design, and Treatments

The experimental materials were cultivated under controlled greenhouse conditions at Hainan University, Sanya, China. The study focused on *P. cablin* plants, which were subjected to a sterilization protocol before cultivation. Specifically, patchouli cuttings were immersed in a fungicidal carbendazim solution at 1 g·L^−1^ concentration for 5–8 min. After sterilization, the cuttings were maintained in double-distilled water (ddH_2_O) until root initiation was observed. Following initial rooting, the seedlings were transplanted into cultivation containers (25 cm long, 16.5 cm wide, 13.5 cm high), each filled with 4 L of Hoagland nutrient solution. This study adopted a hydroponic system for experiments to achieve precise regulation of salt stress factors, homogenization of experimental samples, and dynamic monitoring of plant phenotypic changes. Environmental parameters were rigorously controlled throughout the experiment, with temperatures maintained between 24 °C and 26 °C and a photoperiod of 16 h light and 8 h dark, consistent with established protocols [33]. According to the prior screening experiments (Figure S1), it was found that under the treatment of 50 mM NaCl, the seedlings can survive and exhibit obvious phenotypes. The GSH solutions were prepared for the experimental treatments, with a 1 mM GSH concentration selected based on preliminary trials and prior research [34]. The experimental design encompassed four treatment groups: (1) CK: control (untreated); (2) G: 1 mM GSH; (3) S: 50 mM NaCl; and (4) GS: 1 mM GSH + 50 mM NaCl. After a 7-day acclimation period, before initiating salt stress, Groups G and GS received three glutathione spray pretreatments at 1-day intervals. Spraying ensured uniform wetting of both leaf surfaces until the solution adhered without dripping. CK and S groups received equal volumes of distilled water as controls during the corresponding periods. After three rounds of pretreatments, the S and GS groups received a 50 mM NaCl solution to induce salt stress. Phenotypic observations and physiological parameter measurements were conducted on day 8 post-salt stress initiation.

### 2.2. Measures of Growth Parameters and Relative Water Content

Growth parameters of patchouli seedlings were quantitatively assessed through detailed morphological analyses. Measurements included plant height (cm), foliar attributes (such as leaf area (cm^2^) and leaf number (count)), and biomass accumulation in both aboveground and belowground tissues, encompassing stem fresh weight (SFW, g), stem dry weight (SDW, g), root fresh weight (RFW, g), and root dry weight (RDW, g). Leaf surface area was determined using the CI-202 laser scanning system. For biomass evaluation, SFW and RFW were recorded immediately post-harvest, followed by oven-drying at 80 °C for 72 h to obtain dry weight (DW, g) values. Susceptibility indices for SDW and RDW (SDSI and RDSI, respectively) were calculated according to the formula proposed by Kumar et al. [35]:

SDSI/RDSI = [Shoot or Root DW (Treatment)/Shoot or Root DW (Control)] × 100%

Relative water content (RWC) was measured following the protocol outlined by Kumar et al. [35]. To determine turgor weight, leaf samples were hydrated in double-distilled water (ddH_2_O) for 4 h to achieve full turgidity before weighing. Subsequently, the hydrated leaves were dried in an oven at 70 °C until a constant weight was attained.

### 2.3. Analysis of Photosynthetic Pigments

Photosynthetic pigment analysis was performed using 0.10 g of fresh leaf tissue, homogenized in an 80% acetone solution. After centrifugation, the supernatant was collected for spectrophotometric evaluation. Concentrations of photosynthetic pigments were quantified by measuring absorbance at specific wavelengths: 470 nm for carotenoids, 663 nm for chlorophyll a, and 646 nm for chlorophyll b, following the methodology of Perales-Vela et al. [36]. Key photosynthetic parameters, including the maximum quantum efficiency of photosystem II (Fv/Fm) and the quantum yield of photosystem II (Y (II)), were assessed using a PAM-2500 portable chlorophyll fluorometer (Heinz Walz GmbH, Effeltrich, Germany), as described by Lichtenthaler et al. [37].

### 2.4. Analysis of Antioxidant Enzymes and Antioxidants

Biochemical assays were conducted using 0.50 g of frozen leaf samples to evaluate the concentrations of soluble protein, MDA, ascorbic acid (AsA), and glutathione (GSH), as well as the activities of key antioxidant enzymes, namely CAT, SOD, POD, ascorbate peroxidase (APX), glutathione reductase (GR), and glutathione S-transferase (GST). The tissue was homogenized in 5 mL of 0.05 M phosphate-buffered saline (PBS, pH 7.4), followed by centrifugation at 10,000× *g* for 15 min to isolate the supernatant for subsequent analyses. The soluble protein concentration was quantified using the Coomassie Brilliant Blue method with a commercial kit (KMSP-2-W), as Ren et al. outlined [38]. Additional biochemical parameters were measured employing specialized kits from Nanjing Jiancheng Bioengineering Institute (Nanjing, China), including GSH (A006-1), APX (A123-1-1), SOD (A001-1), POD (A084-3-1), CAT (A007-1), GR (A062-1-1), GST (A004-1), and AsA (BC1230).

### 2.5. Polyphenol and Flavonoid Analysis

Polyphenol content was determined using a spectrophotometric method. Leaf tissue samples (1 g) were homogenized in a 60% ethanol solution. For extraction, 250 μL of the homogenate was combined with 1.25 mL of 10% Folin–Ciocalteu reagent and 1 mL of 7.5% sodium carbonate solution. Following incubation, absorbance was measured at 765 nm, and total polyphenol content was quantified based on a gallic acid standard curve, expressed as gallic acid equivalents per gram of fresh weight (GAE/g) [39]. Flavonoid content was analyzed by mixing 500 μL of the sample extract with 2 mL of deionized water and 250 μL of 5% sodium nitrite solution. After the designated incubation period, absorbance was measured at 510 nm. Flavonoid concentrations were determined using a catechin standard curve and expressed as catechin equivalents per gram of sample (CAE/g) [40].

### 2.6. RNA Extraction and Transcriptome Sequencing

Total RNA was extracted from leaf tissues using the RNA Easy Fast Tissue Kit (Tiangen, Beijing, China) following the manufacturer’s instructions. RNA quality was assessed using an ultra-micro UV-Vis spectrophotometer (Miulab, Hangzhou, China) and verified via gel electrophoresis. Samples meeting quality standards were either subjected to RNA sequencing (RNA-seq) or stored at −80 °C for future analysis. Transcriptome sequencing of *P. cablin* was performed by Tsingke Biotechnology Co., Ltd. (Beijing, China). Sequence alignment was conducted using HISAT2 against the 1.94 Gb chromosome-level reference genome (GWHBAZF00000000) [2]. Differentially expressed genes (DEGs) between experimental groups were identified using the DESeq2 R package (version 1.26.0), with statistical significance thresholds set at a false discovery rate (FDR) < 0.01 and |Fold Change| ≥ 2. Functional annotation of DEGs was carried out using Gene Ontology (GO) classification and Kyoto Encyclopedia of Genes and Genomes (KEGG) pathway enrichment analysis, implemented via the GOseq R package and KOBAS software (version 2.0), respectively. Weighted gene co-expression network analysis (WGCNA) was performed using the WGCNA R package, with network visualization and analysis conducted in Cytoscape (version 3.6.0, Hill St, San Diego, CA, USA). Analysis parameters included a module similarity threshold of 0.9, a soft-thresholding power of 6, and a minimum module size of 30 genes [41]. Correlations between module eigengenes (MEs) and phenotypic traits were analyzed using Spearman’s rank correlation coefficients. For multi-class categorical traits, one-way ANOVA was used to compare ME values across groups, followed by Tukey’s HSD post hoc tests. Significance of correlations was assessed via two-tailed *t*-tests, with *p*-values adjusted using the Benjamini–Hochberg (BH) procedure to control the false discovery rate (FDR). Modules with FDR < 0.05 were considered significantly correlated with the phenotype. Hub genes within significant modules were defined as genes with module membership (MM) > 0.8 and gene significance (GS) > 0.5. The co-expression network analysis incorporated eight physiological parameters and their associated gene expression profiles.

### 2.7. Quantitative Real-Time PCR (qRT-PCR) Analysis

Quantitative real-time PCR (qRT-PCR) was performed using the Bio-Rad Mx3000P system with ChamQ Universal SYBR qPCR Master Mix (Vazyme, Nanjing, China). The 18S rRNA gene (GenBank: KP694234.1) was used as an internal reference, and the specific qRT-PCR primers are listed in Table S1. The reaction conditions and thermal cycling parameters are detailed in Tables S2 and S3. qRT-PCR was performed in a 20 μL reaction volume containing 10 μL of ChamQ Universal SYBR qPCR Master Mix, 0.4 μL of forward primer (10 μM), 0.4 μL of reverse primer (10 μM), 0.5 μL of template cDNA, and 8.7 μL of nuclease-free water. The thermal cycling protocol included a preincubation step at 95 °C for 10 min (1 cycle), followed by 40 cycles of two-step amplification (95 °C for 10 s, 60 °C for 10 s). A melting curve analysis was then conducted (95 °C for 10 s, 60 °C for 60 s, 95 °C for 15 s; 1 cycle), and the reaction was terminated with a cooling step at 37 °C for 30 s (1 cycle). Relative gene expression levels were calculated using the comparative 2^−ΔΔCt^ method.

### 2.8. Statistical Analysis

All experiments were conducted with three independent biological replicates (*n* = 3). Data are presented as the mean ± SE. A one-way analysis of variance (ANOVA) was performed to assess statistical differences between samples, followed by Duncan’s multiple range test (*p* < 0.05) using SPSS 25.0. Figures were generated using GraphPad Prism 9.5 and Origin 2021.

## 3. Results

### 3.1. Effects of GSH on Growth Parameters of Patchouli Under Salt Stress

**Figure 1:**
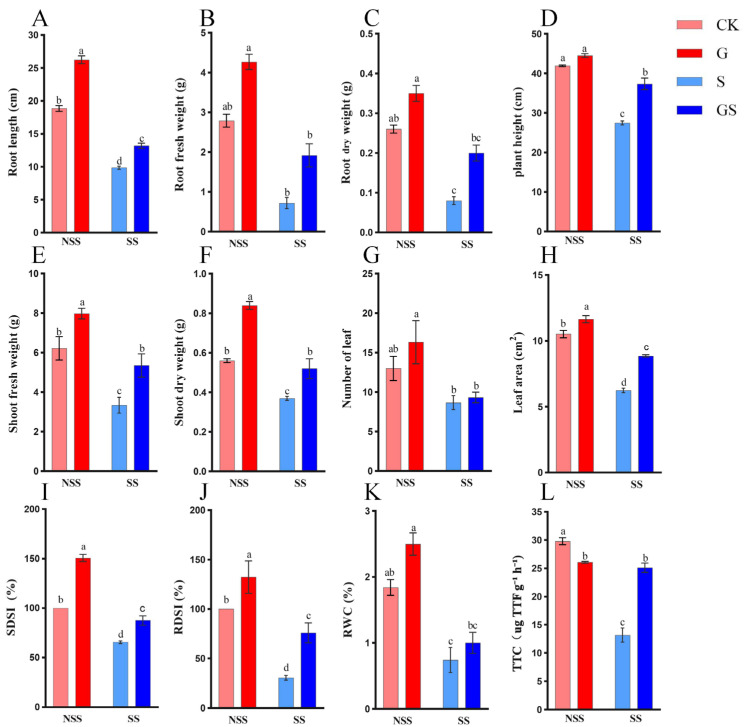
Effects of GSH on the growth parameters of *P. cablin* under salt stress. Different letters indicate statistically significant differences among treatment groups (*p* < 0.05, ANOVA). (**A**) RL, Root length. (**B**) RFW, Root fresh weight. (**C**) RDW, Root dry weight. (**D**) Plant height. (**E**) SFW, Shoot fresh weight. (**F**) SDW, Shoot dry weight. (**G**) Number of leaves. (**H**) Leaf area. (**I**) SDSI, Susceptibility indices for SDW. (**J**) RDSI, Susceptibility indices for RDW. (**K**) RWC, Relative water content. (**L**) TTC, Root activity. Data are presented as mean ± SE. NSS, no salt stress; SS, salt stress; CK, control; G, 1 mM GSH; S, 50 mM NaCl; GS, 1 mM GSH + 50 mM NaCl.

Exposure to 50 mM NaCl significantly reduced the growth parameters of *P. cablin* compared with the control (CK), including plant height (34.5%), root fresh weight (RFW, 74.2%), root dry weight (RDW, 69.2%), shoot fresh weight (SFW, 46.3%), shoot dry weight (SDW, 33.9%), leaf number (33.3%), leaf area (40.7%), and relative water content (RWC, 59.8%) (Figure 1). Pretreatment with GSH, however, had a positive effect on the quality of seedlings under both control (CK) and salt stress (S) conditions (*p* < 0.05). Under normal conditions, GSH treatment significantly improved the growth parameters compared with CK, such as SFW (28.3%), SDW (50.0%), and root length (39.3%). Meanwhile, under salt stress, GSH application markedly improved these growth indicators. These findings suggest that GSH pretreatment enhances the growth and biomass accumulation of *P. cablin* under salinity stress. Furthermore, root activity, a key indicator of root health and function, was greatly influenced by salt stress. Under salt stress, root activity was significantly inhibited by 55.8% compared with the control. However, exogenous GSH supplementation effectively alleviated this inhibitory effect, suggesting that GSH may protect root cells through specific mechanisms, thereby enhancing plant adaptation to salinity. Overall, these results demonstrate that exogenous GSH supplementation mitigates the adverse effects of salt stress and upgrades the quality of *P. cablin* seedlings under both normal and salt conditions.

### 3.2. Effects of GSH on Photosynthetic Pigments, Osmolytes, and Non-Enzymatic Antioxidants Under Salt Stress

**Figure 2:**
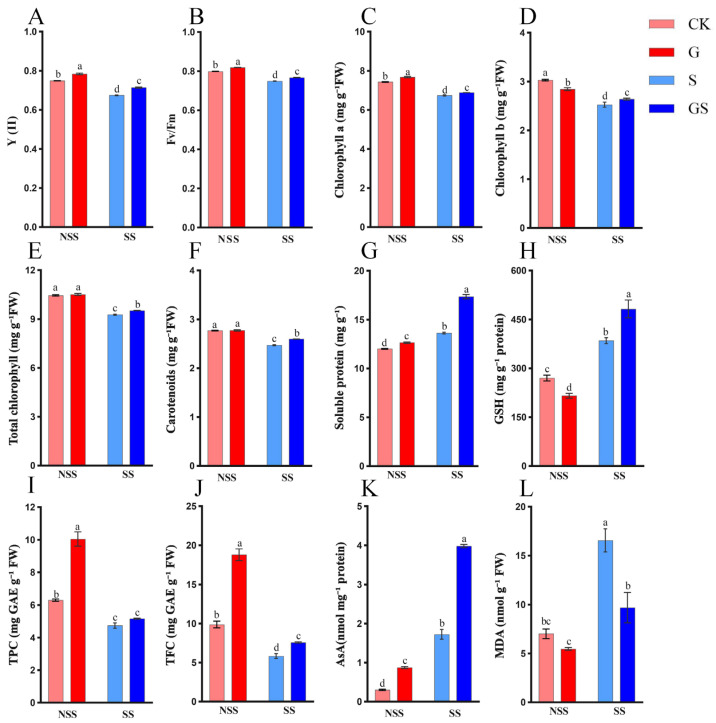
Effects of GSH on photosynthetic pigments and metabolites in *P. cablin* leaves under salt stress. (**A**) Y (II), the quantum yield of photosystem II. (**B**) Fv/Fm, PSII maximum efficiency. (**C**) chlorophyll a. (**D**) chlorophyll b. (**E**) Total chlorophyll. (**F**) carotenoids. (**G**) soluble protein. (**H**) GSH, glutathione. (**I**) TPC, total polyphenols. (**J**) TFC, total flavonoids. (**K**) AsA, ascorbate. (**L**) MDA, malondialdehyde. Different letters indicate statistically significant differences among treatment groups (*p* < 0.05, ANOVA). Data are presented as mean ± SE. CK, control; NSS, no salt stress; SS, salt stress; G, 1 mM GSH; S, 50 mM NaCl; GS, 1 mM GSH + 50 mM NaCl.

Salt stress significantly impacted photosynthetic parameters and pigment composition in *P. cablin* leaves. Compared with the control (CK), exposure to 50 mM NaCl markedly reduced photosynthetic parameters and chlorophyll content (Figure 2A–F). However, pretreatment with exogenous GSH before salt exposure mitigated the decline in photosynthetic pigments, leading to a significant increase in chlorophyll a (2.0%), chlorophyll b (4.6%), carotenoids (5.2%), quantum yield of photosystem II [Y (II)] (5.8%), and PSII maximum efficiency (Fv/Fm) (2.4%) (*p* < 0.05). These results suggest that GSH is protective in preserving chlorophyll integrity under salt stress, thereby maintaining normal photosynthetic function.

Plants employ both osmotic regulation and non-enzymatic antioxidant defense mechanisms to enhance their resilience to adverse environmental conditions. Under salinity stress, exogenous GSH application significantly increased the contents of soluble protein (27.3%), GSH (25.3%), and ascorbate (AsA) (155.4%) (Figure 2G,H,K), effectively alleviating salt stress-induced oxidative damage and improving the plant’s adaptive capacity. Moreover, MDA content (Figure 2L), a marker of lipid peroxidation, was significantly reduced by 41.4% in GSH-treated seedlings, indicating a protective effect on cell membrane integrity. Furthermore, the contents of total polyphenol and flavonoid were elevated by 8.9% and 30.0% in GSH-pretreated plants (Figure 2I,J), enhancing the antioxidant defense system and improving overall stress tolerance. These findings demonstrate that exogenous GSH provides dual protective benefits by regulating osmotic potential and enhancing non-enzymatic antioxidant defenses, thereby promoting growth stability in *P. cablin* under salt stress conditions.

### 3.3. Effects of GSH on Antioxidant Enzyme Activity

**Figure 3:**
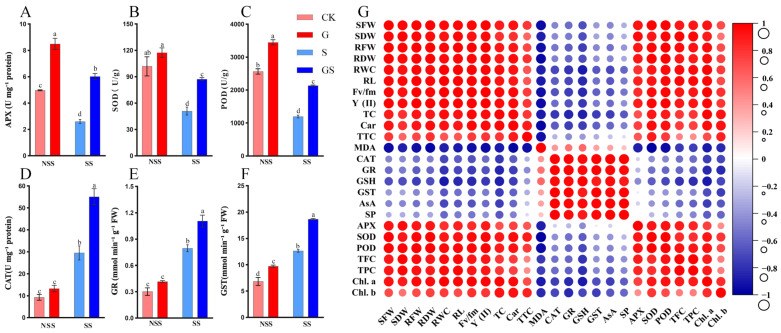
Effects of GSH on antioxidant enzyme activity under salt stress. (**A**) APX, ascorbate peroxidase; (**B**) SOD, superoxide dismutase; (**C**) POD, peroxidase; (**D**) CAT, catalase; (**E**) GR, glutathione reductase; (**F**) GST, glutathione S-transferase. Different letters indicate statistically significant differences among treatment groups (*p* < 0.05, ANOVA). Data are presented as mean ± SE. NSS, no salt stress; SS, salt stress; CK, control; G, 1 mM GSH; S, 50 mM NaCl; GS, 1 mM GSH + 50 mM NaCl. (**G**) Interparameter correlation analysis of growth and physiological-biochemical characteristics in GSH-treated *P. cablin* under saline conditions. The circle size indicates the absolute value of the number.

Salt stress significantly influenced antioxidant enzyme activity in *P. cablin*. Compared with the control (CK), exposure to 50 mM NaCl markedly increased the activities of GST (84.5%), CAT (216.1%), and GR (163.8%) while significantly reducing the activities of POD (53.4%), APX (47.8%), and SOD (50.0%) (Figure 3A–F). However, under salt stress conditions, exogenous GSH supplementation further enhanced the activities of GST (47.6%), CAT (86.7%), and GR (38.8%) while partially restoring APX (132.0%), SOD (71.4%), and POD (78.4%) activities. These findings indicate that GSH-mediated antioxidant defense system regulation effectively alleviates salinity stress-induced oxidative damage. By activating key antioxidant enzymes, GSH enhances ROS detoxification, reduces oxidative damage, and supports maintaining growth performance under salt stress.

### 3.4. Pearson Correlation Analysis

Pearson correlation analysis revealed significant associations among growth parameters, physiological attributes, and biochemical parameters of *P. cablin* in salt stress and exogenous GSH application (Figure 3G). The parameters, including AsA, MDA, CAT, GR, and GST, exhibited positive correlations with salt stress. Meanwhile, growth parameters, root traits, and photosynthetic pigment levels displayed negative correlations with salt stress. In contrast, GSH pretreatment was positively associated with growth parameters, photosynthetic characteristics, osmotic adjustment compounds, antioxidants, and enzymatic activities under salinity stress conditions. These adaptive responses collectively contributed to enhanced growth performance and seedling quality and improved salinity tolerance in *P. cablin*.

### 3.5. Transcriptomic Data Analysis

Transcriptomic profiling was performed on *P. cablin* samples subjected to three different treatments: control (CK), 50 mM NaCl (S), and 1 mM GSH + 50 mM NaCl (GS). Sampling was conducted at two time points (6 h and 24 h) for the S and GS treatments. Quality control of the raw sequencing data yielded 99.38 Gb of clean reads, with an average Q30 score exceeding 97.44% (Table S4), indicating high sequencing accuracy and suitability for subsequent bioinformatics analyses. To assess sample reproducibility based on gene expression levels, Principal Component Analysis (PCA) and Pearson correlation coefficient analysis were performed (Figure S2). The results demonstrated strong clustering patterns and high correlation coefficients among triplicate samples within each treatment group. In contrast, control (CK) and treated samples exhibited distinct spatial separation and lower correlation indices, reflecting significant transcriptomic divergence across experimental conditions. These findings confirm the high reproducibility of biological replicates and the reliability and stability of the transcriptomic dataset for further analysis.

### 3.6. Identification and Enrichment Analysis of Differentially Expressed Genes

**Figure 4:**
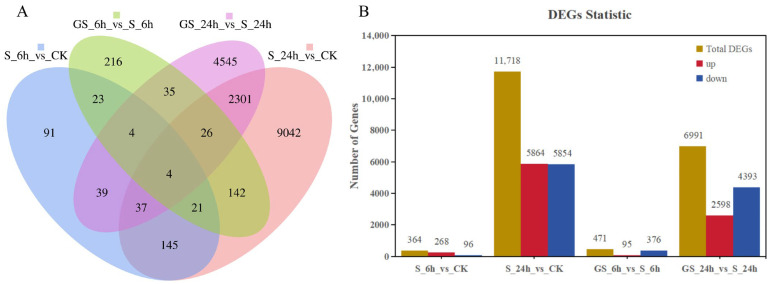
Identification and expression analysis of DEGs. (**A**) Venn diagram illustrating the overlap of DEGs among different treatment groups. (**B**) Statistical distribution of DEGs across comparison groups.

A total of 16,671 differentially expressed genes (DEGs) in *P. cablin* leaves were identified across four comparison groups using a screening threshold of FDR < 0.01 and |Fold Change| ≥ 2 (Figure 4 and Table S5). Among these, 207 DEGs were commonly regulated at both 6 h and 24 h under salt stress, while 69 DEGs were shared across both time points in the GSH + salt stress. Notably, only 4 DEGs were consistently differentially expressed across all four comparison groups. The number of unique DEGs for each comparison was 91, 9042, 216, and 4545, respectively (Figure 4A). Further analysis of individual comparisons revealed that in the S-6 h vs. CK group, 364 DEGs were identified, with 268 upregulated and 96 downregulated. In the S-24 h vs. CK group, 11,718 DEGs were detected, with 5864 upregulated and 5854 downregulated. In the GS-6 h vs. S-6 h group, 471 DEGs were identified, including 95 upregulated and 376 downregulated genes. In the GS-24 h vs. S-24 h group, 6991 DEGs were discovered, with 2598 upregulated and 4393 downregulated genes (Figure 4B). These results emphasize the dynamic transcriptomic responses of *P. cablin* to salt stress and the qualifying effects of GSH treatment.

The DEGs were subsequently annotated and functionally enriched using the Gene Ontology (GO) database (Figure S3). A total of 18,145 DEGs from the S_6 h_vs._CK group, 9484 DEGs from the S_24 h_vs._CK group, 9484 DEGs from the GS_6 h_vs._S_6 h group, and 9484 DEGs from the GS_24 h_vs._S_24 h group were successfully mapped to GO annotations. The DEGs were classified into three major categories based on their functional classifications. Within the biological process category, 100, 3335, 122, and 2261 DEGs were associated with metabolic processes in the four respective comparison groups (Figure S3). In the molecular function category, 99, 3359, 111, and 2431 DEGs were linked to catalytic activity. The significant enrichment of DEGs in both biological processes and molecular functions suggests that GS and S treatments profoundly influence metabolic pathways and catalytic activity in *P. cablin*. Notably, the regulatory effects of GSH in response to salt stress were more pronounced at the 24 h time point.

**Figure 5:**
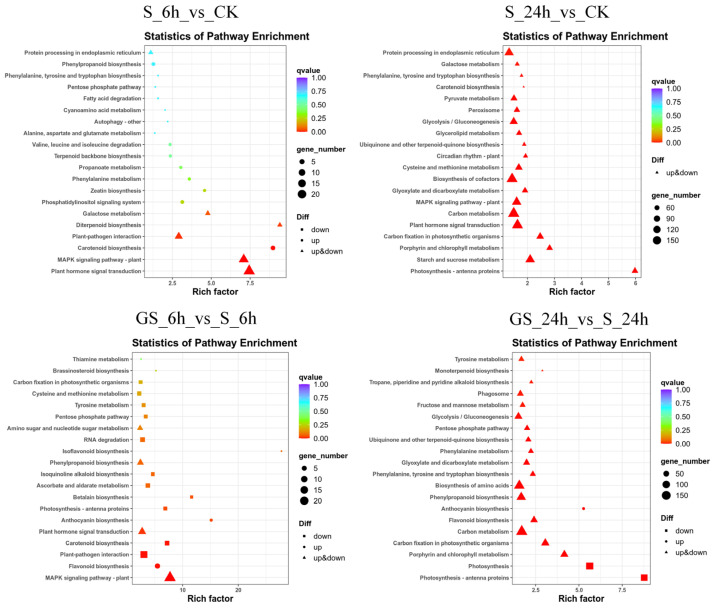
KEGG enrichment analysis of DEGs across different treatment groups.

To evaluate the effects of GSH treatment on secondary metabolite biosynthesis in *P. cablin*, pathway enrichment analysis was conducted using gene set enrichment analysis (GSEA) and the KEGG database. The results identified 66, 2233, 82, and 1650 DEGs associated with 33, 131, 37, and 126 metabolic pathways in the S_6 h_vs._CK, S_24 h_vs._CK, GS_6 h_vs._S_6 h, and GS_24 h_vs._S_24 h groups, respectively. In the 6-h comparison groups for all treatments, DEGs were predominantly developed in the MAPK signaling pathway in plants, plant-pathogen interactions, and plant hormone signal transduction. However, in the 24 h comparison groups, DEGs were primarily associated with carbon metabolism and amino acid biosynthesis (Figure 5). Further, GSEA on KEGG pathway-associated DEGs revealed that three DEGs in the S_6 h_vs._CK group were augmented in the phenylpropanoid biosynthesis pathway, all of which were upregulated, including *Pat_B17G151400* (phenylalanine ammonia-lyase), *Pat_A17G009100* (beta-glucosidase BoGH3B-like), and *Pat_A17G168400* (phenylalanine ammonia-lyase) (Figure S4). In the S_24 h_vs._CK group, DEGs were enriched in the terpenoid backbone biosynthesis pathway, with 43 genes identified, 32 of which were upregulated. Key upregulated genes included *GGPS* (geranylgeranyl pyrophosphate synthase), *ACAT* (acetyl-CoA acetyltransferase), *DXPS* (1-deoxy-D-xylulose-5-phosphate synthase), *HMGCR* (3-hydroxy-3-methylglutaryl-coenzyme A reductase), *HDR* (4-hydroxy-3-methylbut-2-enyl diphosphate reductase), and *SPS* (solanesyl-diphosphate synthase 3). The GS_6 h_vs._S_6 h comparison revealed seven significantly upregulated DEGs enriched explicitly in the flavonoid biosynthetic pathway. Additionally, this group exhibited enrichment in the anthocyanin biosynthesis pathway with two upregulated genes (*Pat_A12G148000* and *Pat_A11G157700*). Analysis of DEGs following salt stress treatment indicated significant enrichment in secondary metabolite biosynthetic pathways, particularly terpenoid backbone and phenylpropanoid biosynthesis, with most core genes showing an upregulated trend. This suggests that salt stress induces the accumulation of metabolites such as terpenoids as a defense mechanism, albeit at the potential cost of normal growth and development. In contrast, under salt stress conditions, GSH treatment led to the preferential enrichment of DEGs in flavonoid and anthocyanin biosynthetic pathways. This suggests that GSH enhances secondary metabolism, particularly flavonoid accumulation, thereby improving salt tolerance in *P. cablin*.

### 3.7. Weighted Gene Co-Expression Network Analysis (WGCNA) of Transcriptomic and Phenotypic Data

**Figure 6:**
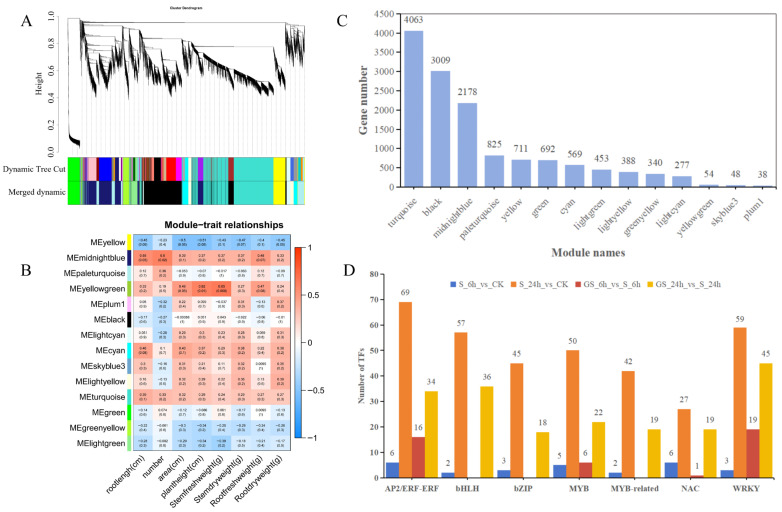
WGCNA of transcriptomic and phenotypic data. (**A**) Hierarchical clustering dendrogram of gene modules. This figure is divided into three steps (from top to bottom). The first part presents a gene system clustering tree based on TOM, illustrating the clustering relationships of each gene. The second part corresponds to the results of the first part, using a dynamic programming algorithm to partition genes into modules. The third part optimizes and merges the results from the second part, yielding the final module partitioning results. (**B**) Heatmap illustrating Pearson correlation coefficients between 14 module eigengenes and 8 physiological traits. (**C**) Gene counts distribution across modules. (**D**) Statistical representation of transcription factors (TFs) within modules.

To further investigate the response of patchouli to GSH under salt stress, WGCNA was executed by integrating transcriptomic data with eight growth parameters, including plant height, root length, root dry weight (RDW), shoot fresh weight (SFW), shoot dry weight (SDW), root number, root fresh weight (RFW), and root area. A total of 13,645 transcripts (FPKM ≥ 1, variation in FPKM: cv ≥ 0.5) were subjected to power value testing, revealing that correlation coefficients remained above 0.7 for power values ranging from 6 to 30, indicating robust connectivity (Figure 6A and Figure S5). Ultimately, 14 distinct co-expression modules were identified (Figure 6B,C). The midnightblue module, containing 2081 genes, displayed a statistically substantial positive correlation (*p* ≤ 0.05) with root length and leaf number and positive associations with other phenotypic traits. Notably, genes within this module exhibited peak expression levels at 24 h following GS treatment (Figure S6A). Functional annotation of the midnightblue module identified 361 genes mapped to the KEGG database (Figure S6B). Specifically, 22 genes were associated with the phenylpropanoid biosynthesis pathway (ko00940), 9 with flavonoid biosynthesis (ko00941), and 4 with α-linolenic acid metabolism (ko00592). Key genes within this module included *4CL*, *COMT1*, *PAL*, peroxidase, *HST*, *UGT*, *CHI*, *FHT*, *CHS*, *AOC*, *LOX2*, and *AOS*, with respective numbers of 1, 1, 1, 6, 4, 1, 4, 1, 2, 1, 2, and 1. Among these, *Pat_A32G099300* was annotated as *4CL*, *MSTRG.92692* as *PAL*, *Pat_A23G101700*, *Pat_B23G083400*, *Pat_A24G091600*, and *Pat_B24G082200* as *CHI*, *Pat_A19G042200* and *Pat_B20G037900* as *CHS*, and *Pat_A01G128400* as a *UGT73* family gene, potentially involved in flavonoid biosynthesis. Notably, all six genes annotated as *CHI* and *CHS* were significantly upregulated following GS treatment, suggesting that these genes positively regulate the flavonoid biosynthesis pathway mediated by GSH signaling.

### 3.8. Transcription Factor (TF) Analysis

Transcription factors (TFs) are pivotal in regulating gene expression associated with stress response pathways. In this study, a total of 52, 827, 70, and 362 differentially expressed transcription factors (DETFs) were identified in the S_6 h_vs._CK, S_24 h_vs._CK, GS_6 h_vs._S_6 h, and GS_24 h_vs._S_24 h groups, respectively (Figure 6D and Table S6). Compared with salt stress alone, the GS_6 h group exhibited 52 DETFs, including 16 *AP2*/*ERF-ERF* (all downregulated), 6 *MYB* (1 upregulated, 5 downregulated), 1 *NAC* (downregulated), and 19 *WRKY* (all downregulated). In the GS_24 h group, a total of 362 DETFs were identified, comprising 34 *AP2*/*ERF-ERF* (9 upregulated, 25 downregulated), 36 *bHLH* (8 upregulated, 28 downregulated), 18 *bZIP* (11 upregulated, 7 downregulated), 22 *MYB* (9 upregulated, 13 downregulated), 19 *MYB*-related (5 upregulated, 14 downregulated), 19 *NAC* (all upregulated), and 45 *WRKY* (41 upregulated, 4 downregulated). These differentially expressed TFs likely perform crucial roles in the regulatory network of patchouli’s response to salt stress, with GSH treatment significantly modulating their expression patterns.

### 3.9. Analysis of the Flavonoid Biosynthesis Pathway in Patchouli

**Figure 7:**
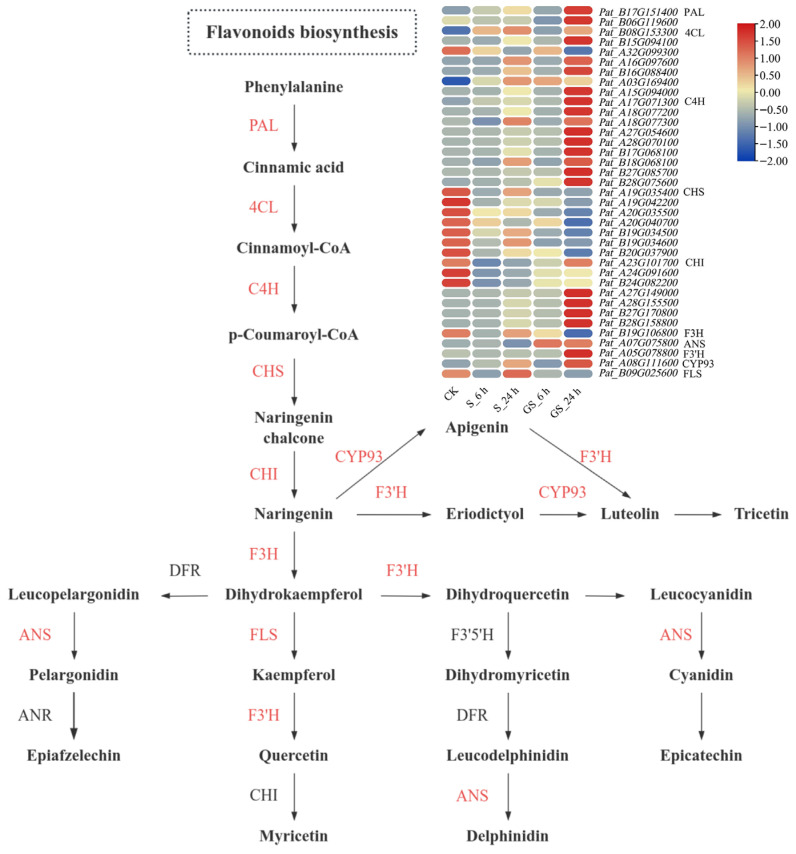
Analysis of key structural genes involved in the flavonoid biosynthesis pathway. The color gradient from blue to yellow to red specifies relative gene expression levels from low to high.

Integrating metabolomic and transcriptomic profiling provides a powerful approach to elucidating key regulatory pathways and identifying functionally significant genes within biological systems. Flavonoids, essential components of the phenylpropanoid pathway, play crucial roles in plant metabolism and stress responses. This study combined transcriptomic data with the known flavonoid biosynthesis pathway to construct a regulatory network of DEGs associated with flavonoid metabolism (Figure 7). This approach allowed us to evaluate the patchouli reaction to salt stress following GSH treatment. Preliminary quantification of total flavonoid content indicated a significant decline in patchouli subjected to salt stress compared with the CK. However, GSH treatment substantially enhanced flavonoid biosynthesis, as evidenced by a marked increase in flavonoid content in GS-treated plants relative to those exposed to salt stress alone. A total of 37 DEGs involved in the flavonoid biosynthesis pathway were identified through GSEA, and their expression levels were compared across different treatments and time points. Notably, the transcriptional levels of several key genes, including *CHI*, *PAL*, *C4H*, *4CL*, *ANS*, *F3′H*, and *CYP93*, peaked at GS_24 h, suggesting that the upregulation of flavonoid biosynthesis primarily drives GSH-mediated enhancement of salt tolerance in patchouli. Overall, salt stress triggered the activation of protective mechanisms in patchouli, resulting in a slight but statistically insignificant upregulation of some defense-related genes. In contrast, GS treatment significantly promoted the transcriptional activation of flavonoid biosynthesis genes, leading to a substantial increase in flavonoid production and improved adaptation to salt stress.

### 3.10. Correlation Analysis Between qRT-PCR and Transcriptome Sequencing Results

**Figure 8:**
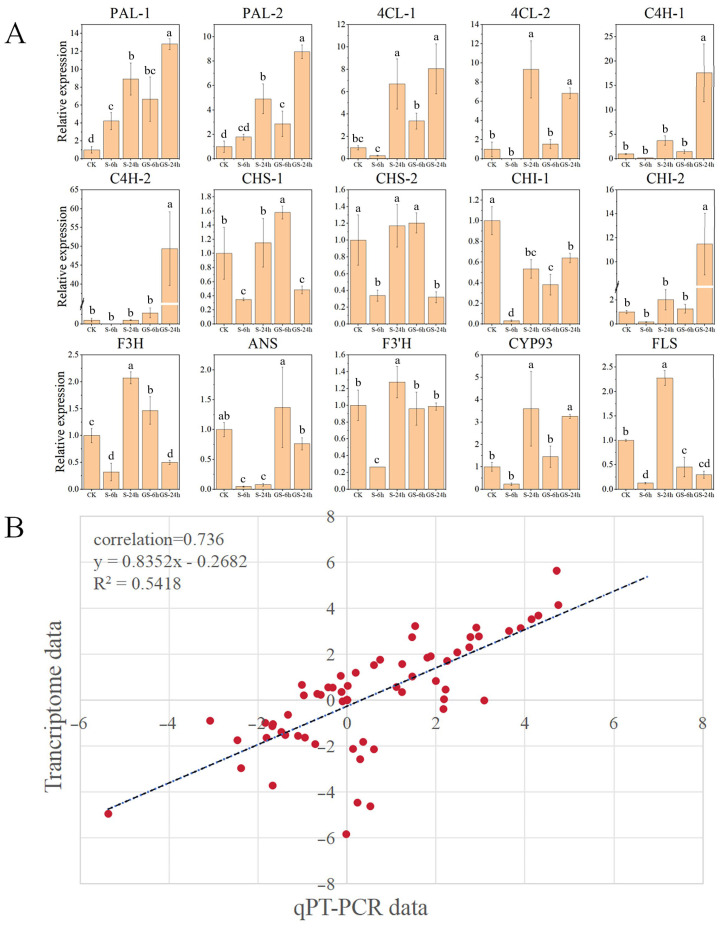
qRT-PCR analysis. (**A**) Relative expression of key structural genes involved in flavonoid biosynthesis. Different letters indicate statistically significant differences among treatment groups (*p* < 0.05, ANOVA). (**B**) A scatter plot with linear regression analysis compares qRT-PCR and transcriptome with Pearson correlation coefficients, indicating the degree of association.

To validate the transcriptomic data, key enzymatic genes involved in flavonoid metabolism (*CHI*, *PAL*, *ANS*, *4CL*, *CHS*, *F3H*, *F3′H*, *CYP93*, *FLS*, and *C4H*) were chosen for qRT-PCR analysis. The patterns of gene expression observed in qRT-PCR closely aligned with the transcriptome sequencing results (Figure 8A). Further comparative analysis demonstrated a significant correlation between the two datasets, with a Pearson correlation coefficient of 0.736, indicating high RNA-seq data reliability and reproducibility (Figure 8B). These findings confirm the accuracy and robustness of the transcriptomic analysis.

## 4. Discussion

### 4.1. GSH-Induced Enrichment of Antioxidant Defense Systems in Patchouli Under Salt Stress

Salt stress leads to excessive soil salinity and oxidative damage in plants, negatively impacting growth, yield, and quality [11,42]. In our study, salt stress inhibited the growth of patchouli stems and roots, significantly reducing fresh and dry biomass (Figure 1). However, pretreatment with 1 mM GSH effectively alleviated salt-induced growth suppression, significantly enhancing biomass accumulation in both shoots and roots and thus promoting the quality of seedlings. Moreover, GSH effectively protected the photosynthetic system against salt-mediated damage via raised chlorophyll a, chlorophyll b, carotenoids, quantum yield of photosystem II [Y (II)], and PSII maximum efficiency (Fv/Fm). The growth-promoting effects of GSH have been consistently demonstrated in various plant species, including *Ipomoea batatas*, *Carthamus tinctorius*, and *Solanum lycopersicum* [17,19,43]. To elucidate the molecular mechanisms for GSH-induced salt stress tolerance in patchouli, this study comprehensively analyzed multiple physiological parameters, including growth performance, photosynthetic efficiency, chlorophyll fluorescence characteristics, osmoregulatory compounds, and antioxidant defense systems.

Salt stress disrupts intracellular ROS homeostasis, leading to excessive ROS accumulation and subsequent oxidative damage in plant cells [14]. This ROS imbalance triggers membrane lipid peroxidation, impairing cellular integrity and function. Enhancing antioxidant enzyme activity to scavenge ROS is an essential defense strategy to mitigate oxidative stress-induced lipid damage. This present study revealed significant changes in oxidative stress markers under salinity stress, including increased MDA accumulation and CAT activity, accompanied by a decline in SOD and POD activities in patchouli seedlings (Figure 2L and Figure 3B–D). These alterations compromised the plant’s antioxidant defense capacity. However, GSH pretreatment significantly improved antioxidant enzyme activities, as evidenced by enhanced SOD, CAT, and POD activities and a corresponding reduction in MDA content. Pearson correlation analysis revealed significant associations between oxidative stress markers, antioxidant defense components, and growth traits (Figure 3G), providing critical insights into the coordinated regulatory network of GSH-mediated salt tolerance. Notably, malondialdehyde (MDA), a hallmark of membrane lipid peroxidation, exhibited a positive correlation with catalase (CAT) activity. This correlation reflects the plant’s adaptive response to salt-induced reactive oxygen species (ROS) bursts: excessive ROS accumulation triggers membrane damage (elevated MDA) and concurrently upregulates CAT activity to scavenge H_2_O_2_, mitigating further oxidative injury. However, salt stress alone led to a disproportionate increase in MDA (Figure 2L) alongside only a modest rise in CAT activity (Figure 3D), indicating an insufficient antioxidant capacity to counteract ROS overproduction. In contrast, GSH pretreatment enhanced this positive correlation by further boosting CAT activity while reducing MDA content, suggesting that GSH optimizes the balance between ROS generation and scavenging—strengthening the plant’s ability to maintain membrane integrity under salt stress. These results indicated that GSH enhances redox homeostasis and strengthens the antioxidant defense system against salt stress. Compared with salt treatment, GSH pretreatment did indeed significantly enhance its activity (SOD, POD, CAT, APX, GST, and GR activities increased by 71%, 78%, 86%, 132%, 47%, and 38%, respectively). The differentially expressed genes related to the antioxidant pathway in the transcriptome (such as SOD (Pat_B19G009100), POD (Pat_A03G181500), APX (Pat_B18G082900), GST (Pat_A27G074600), and GR (Pat_A29G039700) encoding genes) showed upregulation trends (2, 142, 2, 16, and 2 times) in the GS group at 24 h, which is similar to the changes in enzyme activity. Moreover, the expression patterns of these genes are also identical to those of flavonoid synthesis genes (Figure 7), suggesting that they may be co-regulated by common GSH-mediated redox signals. Our findings align with previous studies demonstrating that GSH-mediated activation of antioxidant enzymes plays a pivotal role in ROS scavenging under saline conditions [17,19].

GSH can directly or indirectly mitigate ROS accumulation by acting as a scavenger through the AsA-GSH cycle or via GSH peroxidase, thereby alleviating oxidative stress and improving plant resilience [16,44]. The present study demonstrated that GSH significantly developed the key antioxidant enzyme activities, including APX, GST, and GR (Figure 3A,E,F), which play essential functions in the AsA-GSH cycle. These results suggest that GSH reinforces the plant’s defense mechanisms against ROS accumulation under salt stress conditions. The GSH’s shielding role in mitigating oxidative damage was further supported by its ability to regulate antioxidant enzyme expression and activity [19]. Significant increases in GSH and AsA levels following pretreatment (Figure 2H,K) further enhanced the plant’s antioxidant defense capacity against salt-induced oxidative stress. Exogenous GSH treatment induced an increase in endogenous GSH in plants. Consistent with prior findings, mounted GSH and AsA levels have alleviated oxidative stress in *I. batatas* and *Capsicum annuum* in salt stress conditions [18,44]. Overall, GSH mitigates salt-induced oxidative damage and enhances patchouli’s overall stress resistance by providing multiple layers of protection, thereby supporting plant growth under adverse environmental conditions.

Compared to other exogenous treatments, GSH is more effective in preserving patchouli’s bioactive components: it not only alleviates salt stress but also increases total polyphenol and flavonoid contents (key bioactive components), whereas chemical regulators like strigolactone prioritize growth in *Salvia nemorosa* [42] over secondary metabolite accumulation. Salt stress harms patchouli growth by reducing photosynthetic efficiency and leading to excessive ROS accumulation, which disrupts normal cellular functions. Exogenous GSH pretreatment, however, effectively mitigates salt-induced growth inhibition.

### 4.2. GSH Enhances Salt Tolerance by Regulating Flavonoid Metabolism and Hormone Signaling in Patchouli

Flavonoids, as significant plant secondary metabolites, play crucial roles in various physiological processes, including defense mechanisms, signaling pathways, and oxidative stress mitigation via potent free radical scavenging [45]. When plants encounter environmental stressors such as salinity, ultraviolet radiation, and drought, flavonoid biosynthesis is upregulated, thereby enhancing stress resistance [38,46,47]. In our study, GSH pretreatment significantly improved the total flavonoid content in patchouli leaves (Figure 2J). And the total flavonoid content is strongly positively correlated with antioxidant enzyme activities (SOD, POD, APX) (Figure 3G), suggesting that flavonoid biosynthesis may contribute to the enhanced antioxidant defense system. The transcriptomic analysis further revealed that, compared with the 6 h treatment group, the 24 h group exhibited involvement of a broader range of metabolic pathways, particularly those associated with cofactor biosynthesis in primary metabolism. The DEGs after GSH treatment were significantly enriched in both flavonoid and phenylpropanoid biosynthesis pathways. These findings suggest that salt stress induces substantial alterations in patchouli’s primary metabolism, increasing the extent of metabolic shifts over time. Concurrently, GSH treatment modulates patchouli’s secondary metabolism under salt stress, particularly by upregulating the phenylpropanoid and flavonoid biosynthetic pathways, thereby conferring enhanced salt stress tolerance.

In addition, GSH exerts profound potential influences on plant phytohormone signaling pathways through modulating cellular redox homeostasis and direct/indirect crosstalk with key signaling components [48]. Transcriptomic studies reveal that biosynthesis of abscisic acid, auxin, and jasmonic acid, along with their signaling genes, is activated during GSH treatment [49]. Glutathione enhances cucumber resistance to gray mold by promoting the JA signaling pathway [50]. Regarding abscisic acid (ABA) signaling, the GSH/GSSG redox couple participates in stress response integration by regulating ABA-mediated ROS scavenging and stress memory establishment, as the redox state shift under stress modulates the activity of ABA signaling components such as MAP kinases and bZIP transcription factors [51]. The GS_6 h_vs._S_6 h comparison revealed that this gene set is enriched in the MAPK signaling pathway, which regulates various plant hormone signaling networks and other defense-related pathways. Our analysis identified four genes associated with α-linolenic acid metabolism (ko00592) in the midnightblue module. This suggests that GSH may enhance the expression of JA-related genes in patchouli during salt stress response, thereby improving antioxidant capacity and protecting the plant from oxidative stress induced by salt stress. Overall, these results indicated that GSH may function as a key redox signal integrator, bridging hormone signaling pathways to coordinate plant growth, development, and stress responses.

### 4.3. Flavonoid Biosynthesis Candidate Genes Implicated in Patchouli

This study conducted an in-depth flavonoid biosynthesis pathway analysis in *P. cablin*, identifying key DEGs involved in this metabolic process. Notably, the expression profiles of several genes, including *C4H*, *PAL*, *F3′H*, *4CL*, *ANS*, *CHI*, and *CYP93*, exhibited progressive upregulation with increasing durations of GSH treatment. The protective role of flavonoids in abiotic stress tolerance has been extensively documented, particularly in their ability to modulate ROS metabolism through various mechanisms, including scavenging oxygen radicals and peroxides [52]. What is more, previous studies proved that the coordinated upregulation of key genes in the flavonoid biosynthetic pathway, either individually or synergistically, significantly enhances flavonoid accumulation in plants, thereby improving their stress adaptation capacity. For instance, overexpression of *F3H* and *FLS* in *Nicotiana tabacum* has been shown to elevate total flavonoid content, enhance antioxidant activity, and confer improved salt stress tolerance [53,54]. Interestingly, in *P. cablin*, the transcriptional levels of CHS exhibited a declining trend following salt stress, with a further reduction upon GSH treatment. The transcriptional downregulation of CHS did not lead to a decrease in total flavonoid content (instead, it increased by 30%), suggesting the possibility of post-transcriptional regulation (such as enzyme activity activation) or compensation by other isoenzymes (seven CHS homologous genes were screened, among which six were downregulated and one was upregulated).

When plants are subjected to abiotic stress, they can regulate transcription factors from families such as *MYB*, *bZIP*, and *ERD15* to activate the flavonoid biosynthesis pathway, thereby enhancing stress tolerance. Relevant studies have made progress in species including *Arabidopsis thaliana* [55], *Nicotiana tabacum* [56], and *Glycine max* [57]. In *A. thaliana*, *AtMYB112* and *AtMYB7* interact with flavonoid synthesis genes (*DFR*, *ANS*) to promote anthocyanin accumulation for ROS scavenging under salt stress [55,58]. In *G. max*, *GmbZIP131*, activated by phosphorylation via *SOS2*, upregulates the expression of its target gene *GmICHG*, facilitating isoflavone release and excess ROS scavenging, thus forming the “GmSOS2L-GmbZIP131-GmICHG” salt stress response cascade [57]. Upon MeJA treatment, *GbMYC1* and *GbMYB1* in *Gossypium barbadense* are activated and bind to the promoters of *ANS* and *DFR*, leading to the coordinated upregulation of anthocyanin biosynthetic genes [59]. Similarly, *HY5*, a *bZIP* transcription factor family member, enhances anthocyanin biosynthesis by directly regulating the promoters of multiple structural genes, including *CHI*, *CHS*, *DFR*, and *F3H* [60]. In *P. cablin*, GSH pretreatment may alleviate stress by regulating MYB transcription factors and activating the flavonoid biosynthesis pathway [32]. In this study, GSH pretreatment led to significant transcriptional changes in TFs associated with flavonoid metabolism. Compared with salt treatment alone, one *MYB* transcription factor (*Pat_B08G145300*) showed significantly increased expression at 6 h. At 24 h, the expression levels of nine *MYB* and eleven *bZIP* TFs were significantly upregulated. Notably, the expression levels of *Pat_A10G016800* (*MYB*), *Pat_B15G165500* (*MYB*), *Pat_A22G081400* (*MYB*), *Pat_A21G081600* (*MYB*), *Pat_B22G044300* (*bZIP*), *Pat_A01G196100* (*bZIP*), and *Pat_B31G062000* (*bZIP*) were increased approximately 16-fold, 6-fold, 6-fold, 5-fold, 8-fold, 5-fold, and 4-fold, respectively, compared with salt treatment alone. These structural gene promoters might possess conserved binding sites for *MYB* (MYB recognition element MRE) and *bZIP* (ABA response element ABRE) [29,46]. We therefore hypothesized that these GSH-responsive transcription factors could bind to the promoters of genes involved in flavonoid biosynthesis and drive their expression, although further experimental validation is required to elucidate their precise functional roles.

Based on physiological, biochemical, and transcriptome data from this study, we propose a possible core regulatory framework mediated by GSH to advance the current understanding of GSH-mediated salt tolerance. Salt stress disrupts the ROS-antioxidant balance. Exogenous GSH restores cellular redox homeostasis by directly scavenging ROS and activating the AsA-GSH cycle (increasing APX and GR activities and GSH/AsA content). This redox homeostasis signal further induces the upregulation of transcription factors (such as *MYB*, *bZIP*). These transcription factors directly drive the accumulation of flavonoids (increasing by 30%) by binding to MRE/ABRE motifs in the promoters of flavonoid biosynthetic genes (such as *CHI*, *ANS*), while indirectly restoring the activity of antioxidant enzymes like SOD and POD. This ultimately reduces reactive oxygen species damage (MDA decreased by 42%), thereby enhancing salt tolerance.

## 5. Conclusions

This study advances the current understanding of GSH-mediated salt tolerance by uncovering a species-specific dual mechanism in patchouli: reinforcing antioxidant defenses and activating flavonoid biosynthesis. Salt stress inhibits patchouli growth by impairing photosynthetic efficiency and inducing excessive ROS accumulation that disrupts cellular functions. Exogenous GSH pretreatment effectively alleviates such damage, significantly reducing MDA and ROS levels, protecting the photosynthetic system, and enhancing antioxidant compound contents and enzyme activities. Transcriptomic analysis highlights the central role of the phenylpropanoid metabolic pathway, particularly the flavonoid biosynthetic branch, in facilitating patchouli’s adaptation to salt stress, which is proven by an increase in the contents of total polyphenol and flavonoid and expression of key regulatory genes, such as *PAL*, *4CL*, *C4H*, *CHI*, *ANS*, *F3′H*, and *CYP93*. In salt stress conditions, GSH is a crucial regulator and provides dual defense strategies for modulating flavonoid metabolism and enhancing the plant’s antioxidant defense system. In the future, the further exploration of GSH-flavonoid interactions and their biological functions may uncover key molecular determinants of plant salinity tolerance, offering valuable insights for evolving innovative approaches to boost crop resilience under stress conditions. This study provides rigorous and reliable technical support for clarifying the GSH-mediated mechanism underlying the salt stress alleviation in *P. cablin* and also lays a solid foundation for subsequent field trials, which is of great significance for expanding the cultivation area of *P. cablin* in the future.

## Data Availability

The datasets in this study were uploaded to NCBI, with the accession number PRJNA1238553 accessed on 20 March 2025 (https://www.ncbi.nlm.nih.gov/, Release date: 20 March 2027).

## Supplementary Materials

The following supporting information can be downloaded at: https://www.mdpi.com/article/10.3390/plants15030457/s1, Figure S1: Effects of different salt concentrations on patchouli; Figure S2: Sample PCA and correlation heat map; Figure S3: GO classification plots; Figure S4: GSEA of DEGs highlighting key metabolic pathways influenced by salt stress and GSH treatment; Figure S5: Power value curve of WGCNA; Figure S6: (A) The heatmap of gene expression in midnightblue. (B) KEGG in midnightblue; Table S1: List of qRT-PCR primers for key genes of flavonoid synthesis in *P. cablin*; Table S2: The qRT-PCR reaction system; Table S3: The qRT-PCR reaction procedure; Table S4: List of transcriptome quality control data; Table S5: DEGs in different groups; Table S6: DETFs in different groups.
