# Supplementary material for: Exogenous Glutathione Enhances Salt Tolerance in Patchouli by Promoting the Antioxidant Capacity and Activating the Flavonoid Biosynthesis Pathway
Source: Plants (Basel). 2026 Feb 2;15(3):457. doi: 10.3390/plants15030457 (PMC12899985; doi:10.3390/plants15030457)
Supplement: Supplementary file 1 [file plants-15-00457-s001.zip › supplementary-Figures.pdf]

Supplementary figures

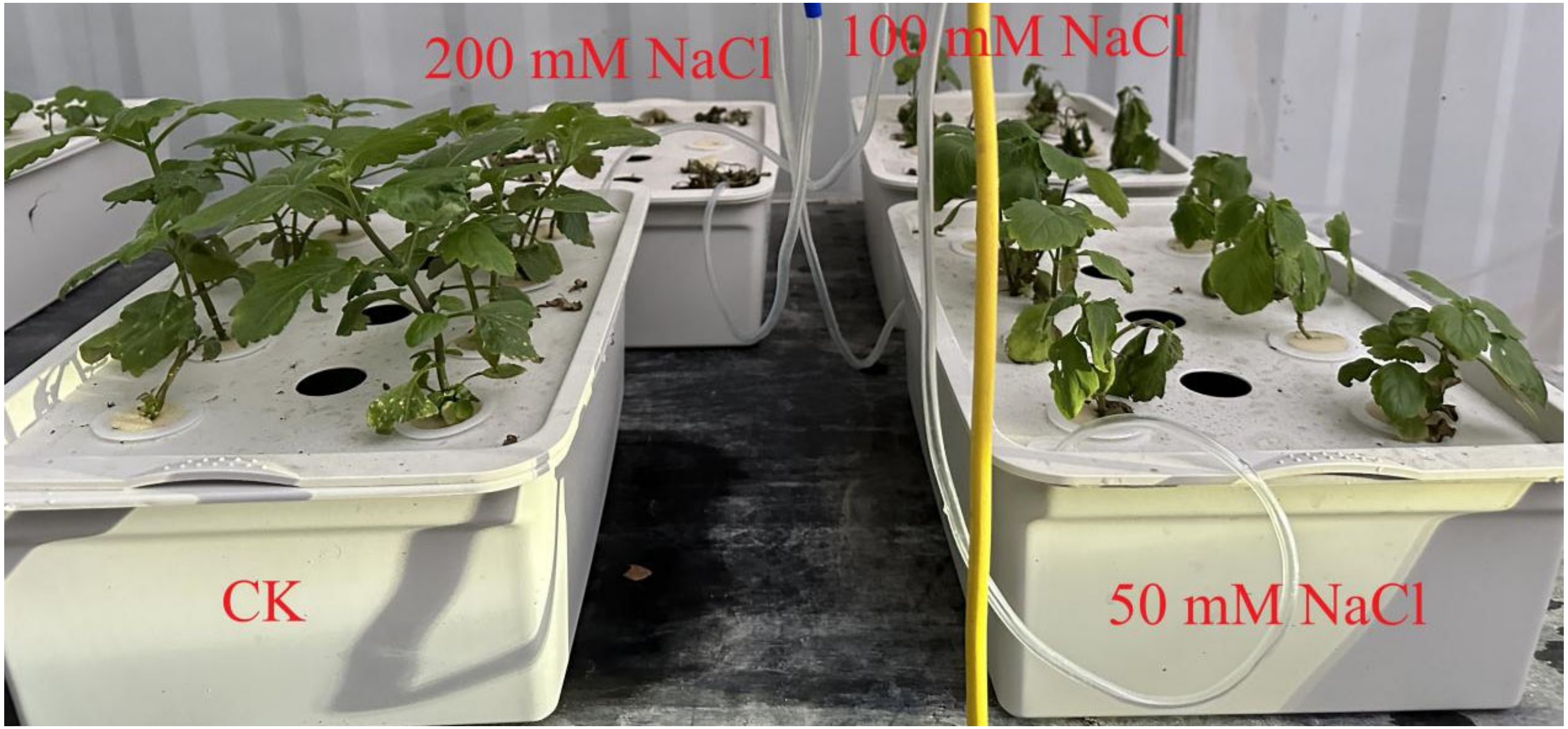

Fig. S1. Effects of different salt concentrations on patchouli.

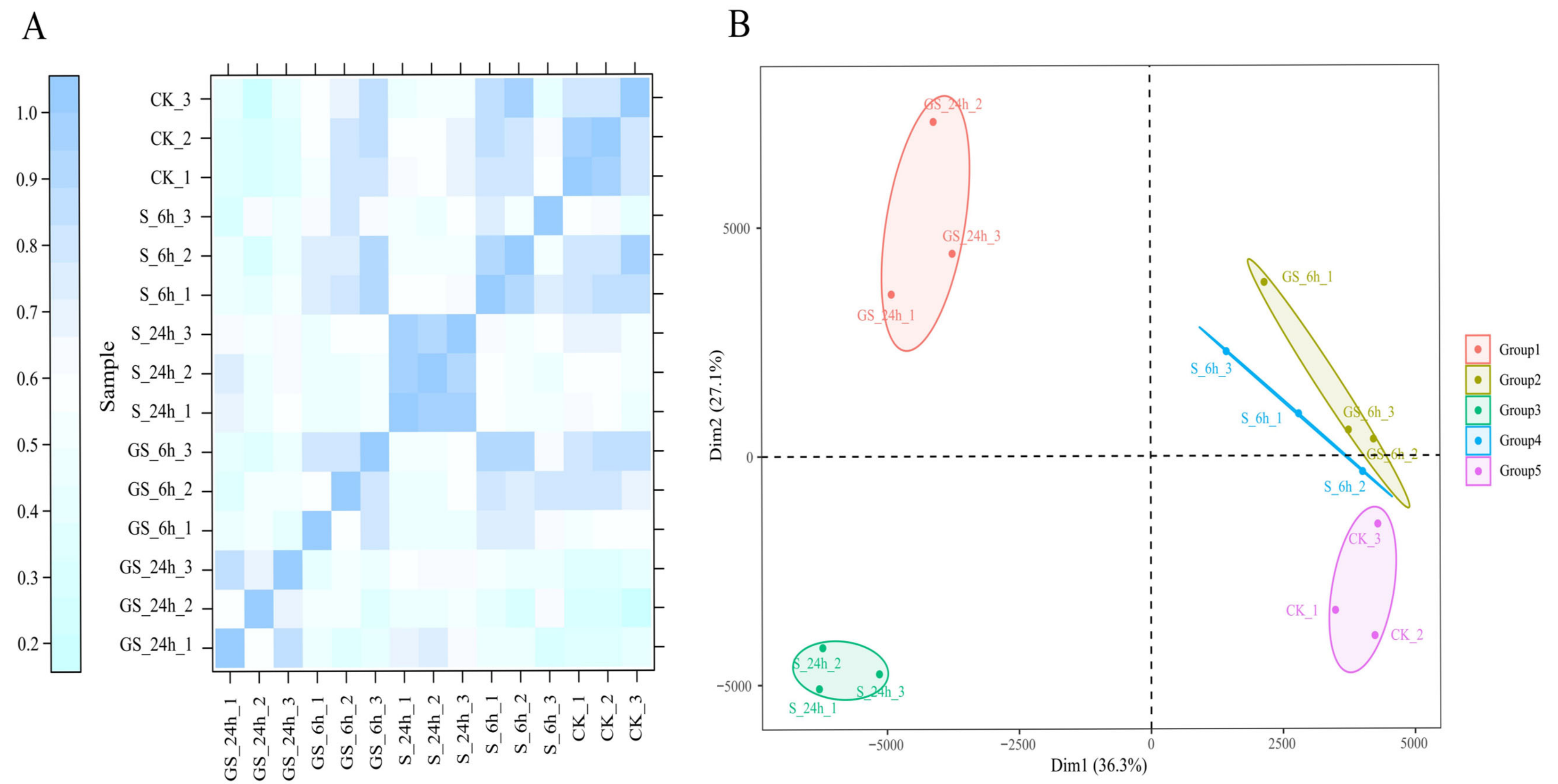

Fig. S2. Sample PCA and correlation heat map.

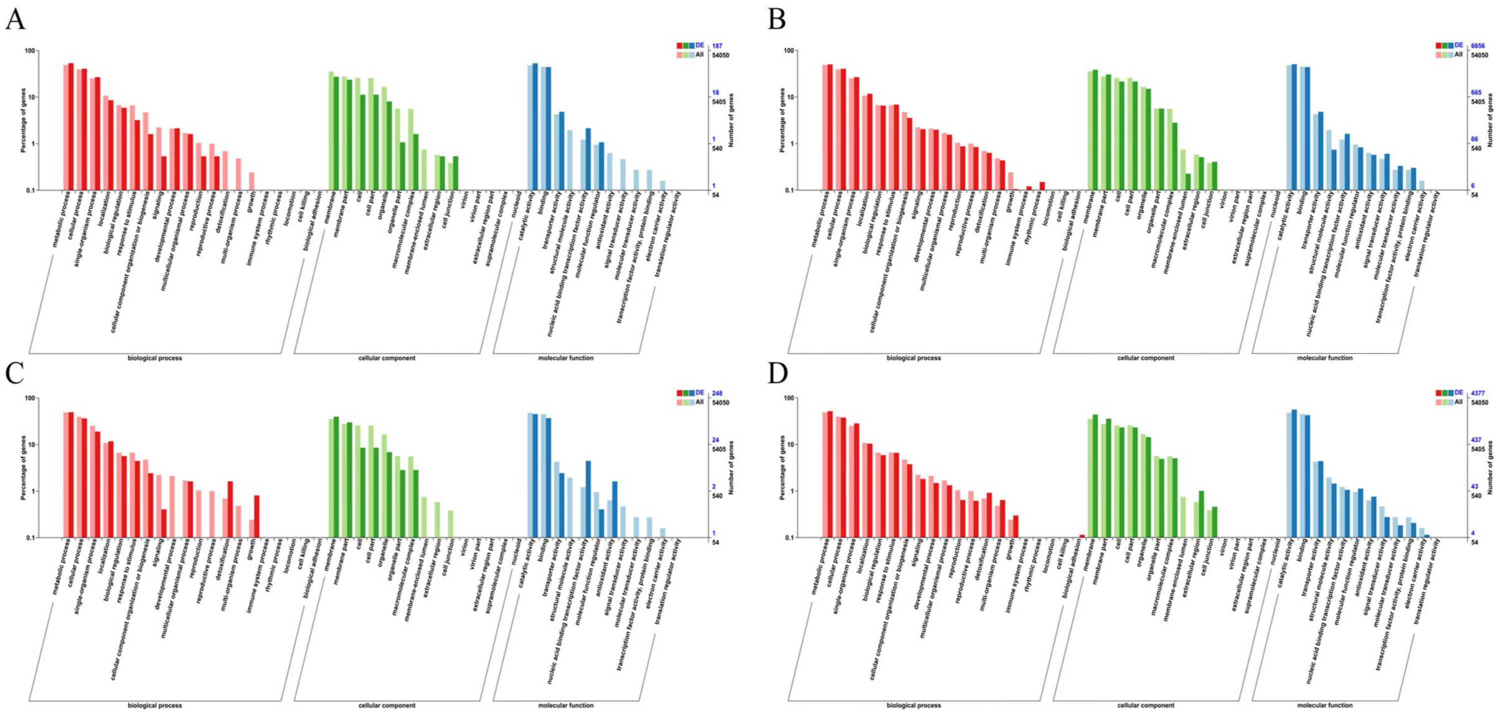

Fig. S3. GO classification plots.

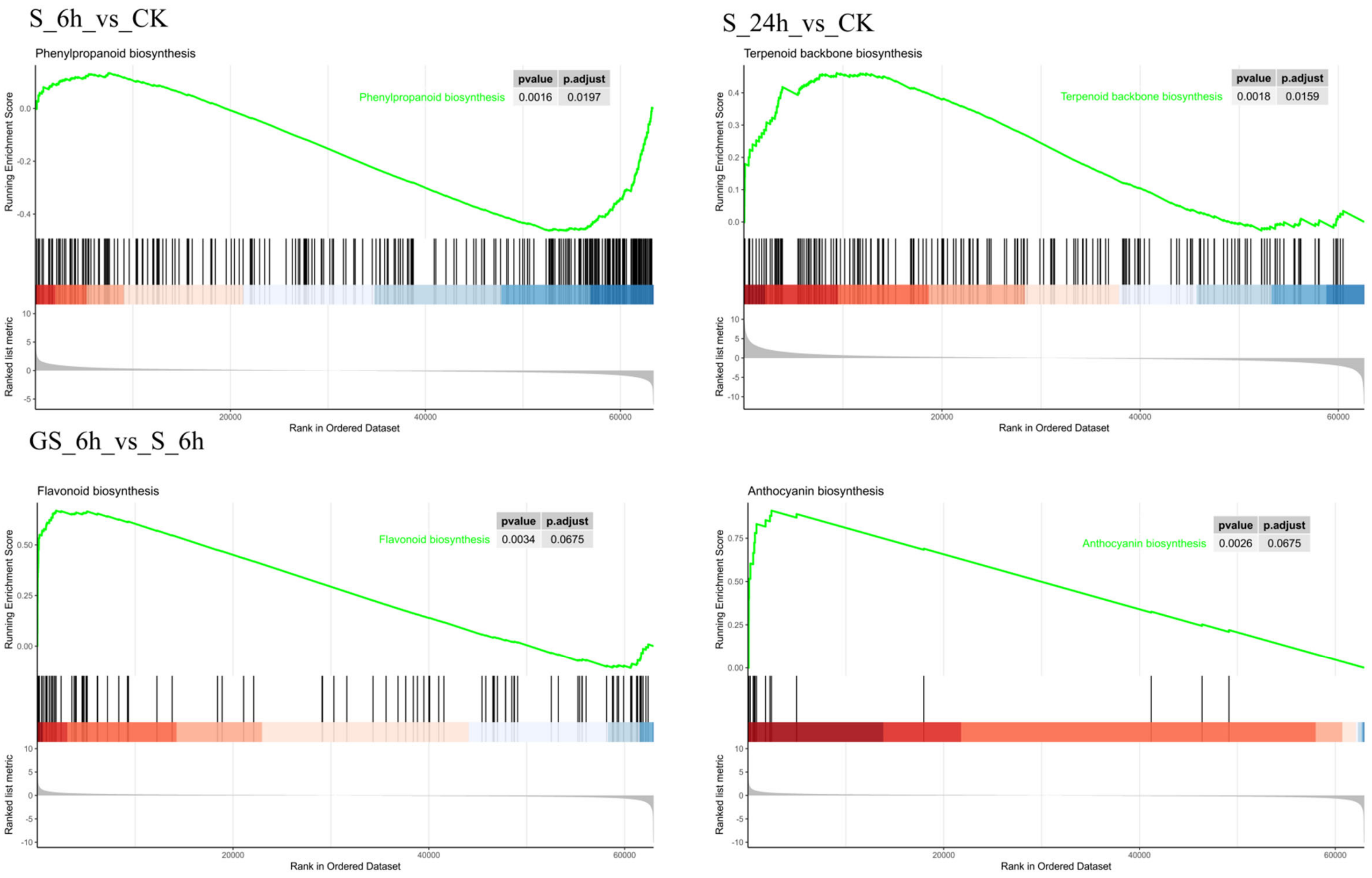

Fig. S4. GSEA of DEGs highlighting key metabolic pathways influenced by salt stress and GSH treatment.

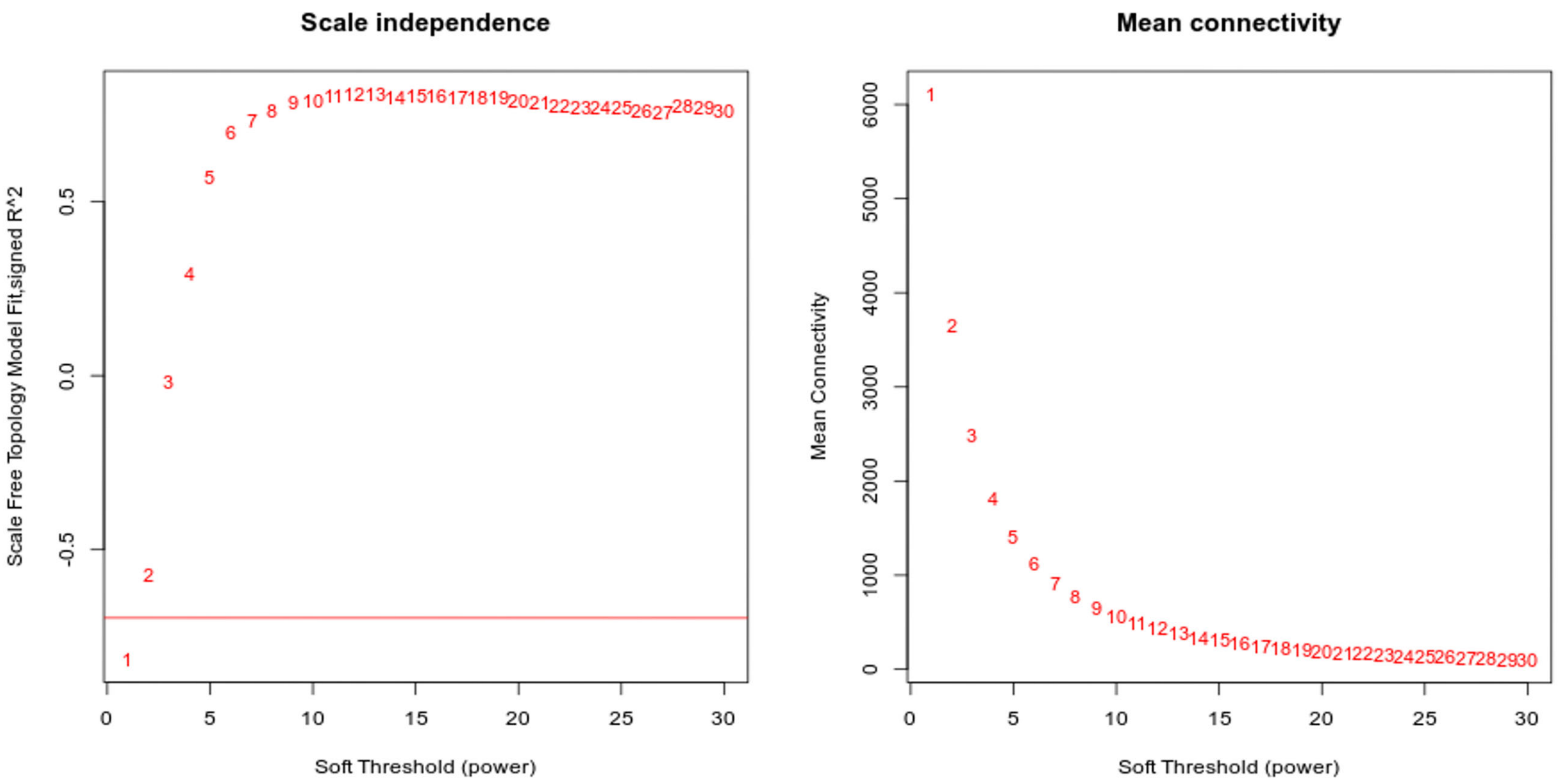

Fig. S5. Power value curve of WGCNA.

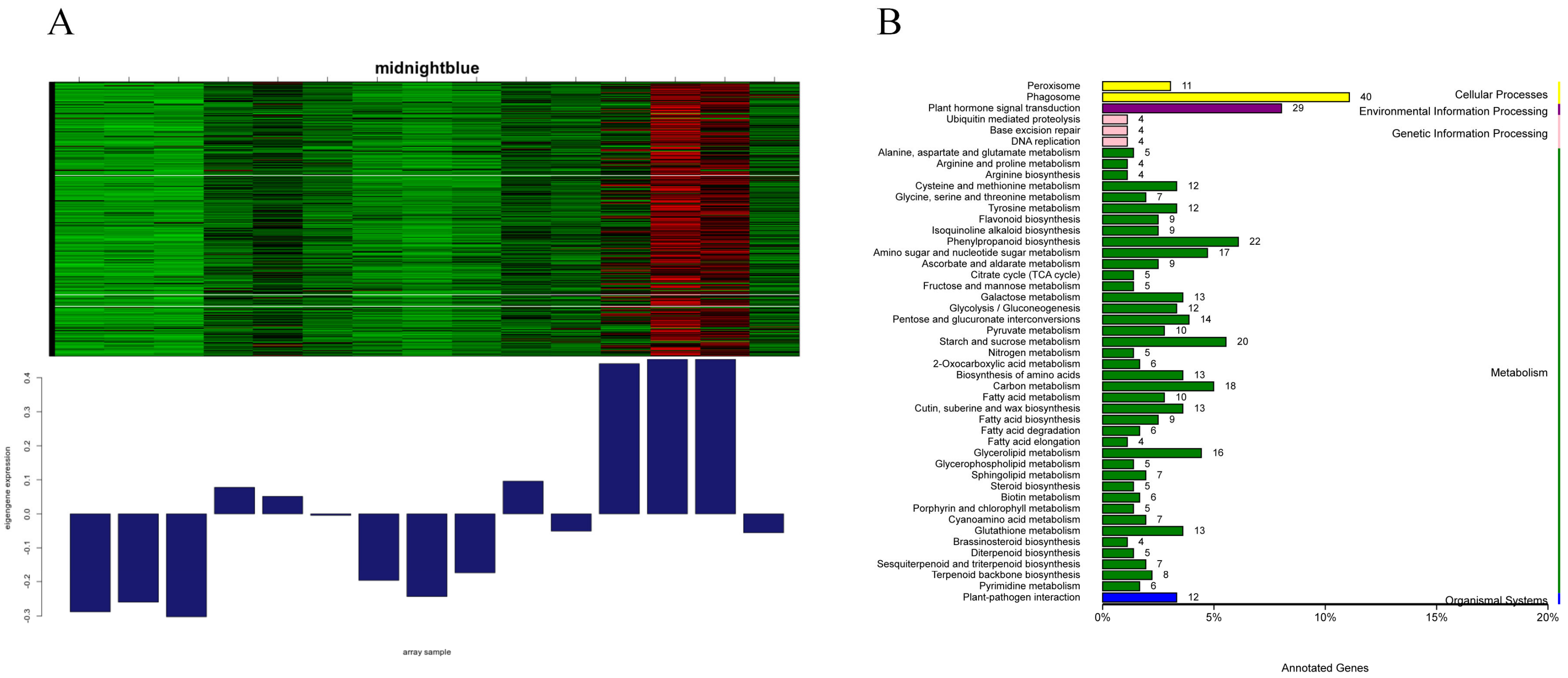

Fig. S6. A. The heatmap of gene expression in midnightblue. B. KEGG in midnightblue.
